# A Systematic Review and Meta-Analysis of the Effects of Food Safety and Hygiene Training on Food Handlers

**DOI:** 10.3390/foods9091169

**Published:** 2020-08-25

**Authors:** Andrea Insfran-Rivarola, Diego Tlapa, Jorge Limon-Romero, Yolanda Baez-Lopez, Marco Miranda-Ackerman, Karina Arredondo-Soto, Sinue Ontiveros

**Affiliations:** 1 Departamento de Ingeniería Industrial, Facultad de Ingeniería, Universidad Nacional de Asunción, Paraguay, San Lorenzo 2160, Paraguay; andrea.insfran@uabc.edu.mx; 2 Facultad de Ingeniería, Arquitectura y Diseño–Universidad Autónoma de Baja California, Ensenada 22870, Mexico; yolanda@uabc.edu.mx; 3 Facultad de Ciencias Químicas e Ingeniería, Universidad Autónoma de Baja California, Tijuana 22390, Mexico; miranda.marco@uabc.edu.mx (M.M.-A.); karina.arredondo@uabc.edu.mx (K.A.-S.); 4 Facultad de Ciencias de la Ingeniería, Administrativas y Sociales, Universidad Autónoma de Baja California, Tecate 21460, Mexico; sinue.ontiveros@uabc.edu.mx

**Keywords:** food safety, hygiene, foodborne diseases, knowledge, attitude, practices, behavior, food handlers, training

## Abstract

Foodborne diseases are a significant cause of morbidity and mortality worldwide. Studies have shown that the knowledge, attitude, and practices of food handlers are important factors in preventing foodborne illness. The purpose of this research is to assess the effects of training interventions on knowledge, attitude, and practice on food safety and hygiene among food handlers at different stages of the food supply chain. To this end, we conducted a systematic review and meta-analysis with close adherence to the PRISMA guidelines. We searched for training interventions among food handlers in five databases. Randomized control trials (RCT), quasi-RCTs, controlled before–after, and nonrandomized designs, including pre–post studies, were analyzed to allow a more comprehensive assessment. The meta-analysis was conducted using the random-effects model to calculate the effect sizes (Hedges’s g) and 95% confidence interval (CI). Out of 1094 studies, 31 were included. Results showed an effect size of 1.24 (CI = 0.89–1.58) for knowledge, an attitude effect size of 0.28 (CI = 0.07–0.48), and an overall practice effect size of 0.65 (CI = 0.24–1.06). In addition, subgroups of self-reported practices and observed practices presented effect sizes of 0.80 (CI = 0.13–1.48) and 0.45 (CI = 0.15–0.76) respectively.

## 1. Introduction

Food safety is a global public health threat with frequent incidents of foodborne diseases. Additionally, the COVID-19 outbreak has put more pressure on global public health; particularly, organizations of producers and providers along the food supply chain are facing an ongoing challenge to improve and to extreme food safety and hygiene due to the pandemic. In this context, foodborne diseases are responsible for major economic costs for a country [1,2]. In terms of global estimates, in 2010, 31 foodborne hazards caused 420,000 deaths and 600 million foodborne illnesses derived from disease agents, such as non-typhoidal *Salmonella enterica*, *Salmonella typhi*, *Taenia solium*, hepatitis A, and aflatoxins, to name but a few [3]. In this regard, the application of the Hazard Analysis and Critical Control Point (HACCP) system can improve food safety; however its strength and success in preventing foodborne illnesses depend on it being applied correctly along with the provision of a sanitary infrastructure and the application of principles of good hygiene practice [4]. Current evidence suggests that a substantial number of foodborne illnesses occur through poor food handling practices of food workers [5,6]. Pathogens may appear in food, for instance, through unsafe farm practices, contamination during manufacturing, packaging, or distributing, or contamination in stores [7,8]. Additionally, food purchases from unsafe sources, inadequate cooking or reheating, holding food at room temperature, cross-contamination, poor personal hygiene, or improper food handling practices frequently contribute to foodborne illnesses [9].

To fight the battle against foodborne diseases, governments have resorted to strategies including food regulations and laws to monitor compliance with food safety standards [10,11,12,13]. Additionally, food companies rely on food safety methodologies, including the food Good Manufacturing Practices (GMP), the Good Agricultural Practices (GAP), the Hazard Analysis and Critical Control Points (HACCP) system, and the ISO 22000 standard to assure the safety of their food products [14,15,16]. In such methodologies, training food handlers in food safety is one of the most effective strategies for preventing foodborne diseases [17].

In an attempt to increase both knowledge and practice on food safety and hygiene, different behavioral theories have been used, including the Health Belief Model, in which an individual will perform a preventive behavior depending on their desire to avoid illness (or if ill, to get well) and the belief that a specific health action will prevent (or ameliorate) illness [18,19]; the KAP model, which assumes that an individual’s behavior or practice is dependent on their knowledge (K) and suggests that the mere provision of information will lead directly to a change in attitude (A) and, consequently, a change in behavior or practice (P) [20]; and the theory of planned behavior (TPB) which focuses on the individual’s intention to perform a given behavior and has been advocated by many researchers for the prediction of determinants of a food handler’s behavior [21,22,23,24,25,26,27].

In this regard, there is an implied assumption that such training leads to changes in behavior based on the KAP model [28]. In other words, training affects knowledge [29] and increased knowledge of correct food hygiene practices may be an important factor in changing behavior [22], i.e., the provision of food safety and hygiene training and the effective enactment of safe food handling practices are important for controlling foodborne illnesses [30,31]. Unfortunately, in most cases, food hygiene training does not translate into positive food handling behaviors [25,30].

In this regard, knowledge, attitude, and practice (KAP) surveys have been used widely. They are representative of a specific population to collect information on what is known, believed, and done in relation to a particular topic [32]. In this sense, several studies use training programs based on KAP as well as TPB with the aim of teaching food handlers how to identify food safety hazards and apply good practices regarding food safety.

Knowledge is accumulated through learning processes (which may involve formal or informal instruction), personal experience, and experiential sharing [33,34,35]. Traditionally, it has been assumed that knowledge is automatically translated into behavior [36], despite studies indicating that this is not necessarily true [37,38]. On the other hand, attitude involves evaluative concepts associated with the way people think, feel, and behave [39]. In the food industry, food handlers must gain knowledge of food safety and be aware of and implement proper food handling practices [40]. Practice refers to how people demonstrate their knowledge and attitude through their actions [41].

Previous studies have analyzed the training interventions and relationship between KAP (knowledge, attitude, and practice) and food safety in environments such as hospitals [42,43,44], colleges [45,46,47], food establishments [48,49,50], restaurants [51,52,53], and houses [54,55,56], among others. Despite the effort made [57,58], further evidence of the effects of training interventions on the knowledge, attitudes and practices toward food safety and hygiene of food handlers from different processes along the food supply chain is needed. To address this gap, we conducted a systematic review and meta-analysis of studies conducting training interventions among food handlers involved in different processes including on farms, in food processing facilities, and in restaurants (i.e., from farm to fork).

## 2. Materials and Methods

**Figure 1:**
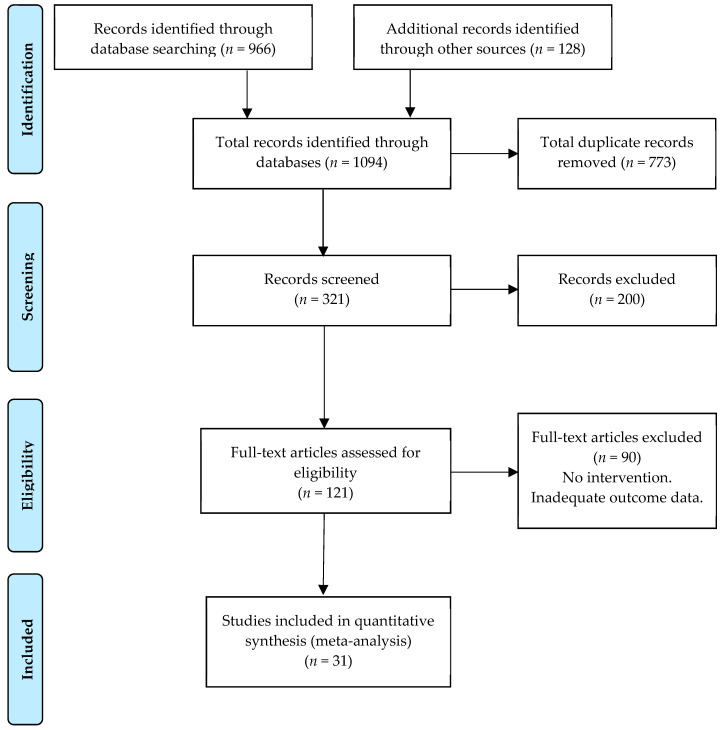
The PRISMA flow chart.

This study adhered closely to the Preferred Reporting Items for Systematic Reviews and Meta-Analyses (PRISMA) guidelines [59,60]. Figure 1 presents a flowchart of the stages involved in the selection process, while the resulting PRISMA checklist summarizes all of the requirements covered (see online Supplementary Table S1). The review was registered in the PROSPERO International Prospective Register of Systematic Reviews (Identifier CRD42019119006).

### 2.1. Search Strategy

We conducted a comprehensive search on the following databases: PubMed, Cochrane Controlled Register of Trials (CENTRAL), Ebsco, Scopus, and Web of Science. Also, we searched for grey literature on Google Scholar and ProQuest. In relation to the search strategy, we relied on both the Peer Review of Electronic Search Strategies (PRESS) [61] and the PICOS (population, intervention, comparator, outcome, and study design) elements. The ultimate search strategy is described in the Supplementary Data S1. We searched for publications in English published between January 1997 and December 2019. Likewise, we examined the reference lists of the retrieved articles to look for further relevant literature. The last search was run in April 2020.

### 2.2. Study Selection

Two authors reviewed the titles and abstracts of the work retrieved during the search. Discrepancies were resolved by discussion and consensus with a third author. All of the reviewed works were conducted among food handlers from different steps of the food supply chain, including farms, food processing facilities, and restaurants (i.e., from farm to fork). Interventions were defined as food safety and hygiene training sessions covering aspects such as personal hygiene, hand washing, cleaning and sanitization, cross-contamination, foodborne diseases, and temperature control. Training was given in the form of talks, demonstrations, self-practice, and different sources of communication, including posters, videos, booklets, slideshows, and fact sheets. We searched for randomized controlled trials (RCTs), quasi-RCTs, and controlled before-after (CBA) studies. In addition, we searched for non-randomized designs, including uncontrolled pre-post studies, to allow a more comprehensive and complete assessment of the available evidence in the area, recognizing that RCTs may not be feasible for many large-scale food safety education interventions [62,63,64,65,66].

The reported food safety training sessions were aligned with regulations, protocols, and guidelines, including, but not limited to, the United Nations’ (UN) Codex Alimentarius, the HACCP, the Food and Drug Administration (FDA) Food Code (including the hand-washing guidelines and protocol), the FDA’s Employee Health and Personal Hygiene Handbook, the United States Department of Agriculture (SDA) Food Safety Education campaign, the European Union General Food Law, Regulation (EC) No. 852/2004, the United Kingdom’s Safety Act, the GMPs, and the Good Hygiene Practices (GHPs). In all of the studies, the comparison group included either participants (i.e., food handlers) who did not receive food safety training or those who had not yet received proper food safety training.

As the main outcomes, all included studies evaluated changes in knowledge, attitude or practice among food handlers. Knowledge refers to the degree of understanding of food handlers about the food safety information given during training sessions. In contrast, attitude refers to a predisposition or tendency to respond positively or negatively to training. Finally, practices are the actions of an individual in response to the knowledge and attitude involved in the training sessions. Similarly, food safety practices can be defined as the increased use of evidence in healthcare practice and policy when both knowledge of, and attitude toward, food safety are present.

Changes in levels of knowledge were measured in the studies through survey-questionnaire data gathered in Likert-type scales with sub-dimensions such as food poisoning, cross-contamination, temperature control, and personal hygiene. Changes in self-reported attitudes toward food safety and hygiene were also measured through survey-questionnaire data on Likert-type scales. Finally, changes in practices were measured, such as self-reported practices and observed practices, the former through survey-questionnaire data in a Likert-type scale and the latter through checklists. Both used different sub-dimensions, including personal hygiene, food safety, and hygiene, temperature control, cross-contamination, sanitation, storage, and food display. We discarded any case report/series and/or review studies with data missing (e.g., sample size, mean, standard deviation), as well as studies conducted among people other than food handlers (e.g., consumers and food transporters).

### 2.3. Data Extraction and Quality Assessment

Two independent reviewers screened each potential article to identify its abstract, title, keywords, and concepts reflecting both the article’s contribution and the research context. Disagreements were overcome by discussion. Then, the relevant full-text studies were retrieved and independently assessed by two reviewers against the review’s inclusion/exclusion criteria. Once more, disagreements were overcome by discussion and consensus with a third author. The data were extracted by one reviewer and checked by a second reviewer. The extracted raw data from each study included authors’ names, year of publication, country of origin, title, study setting, study length, study aim, study design, study population, participant demographics, details on the training interventions and control conditions, recruitment and study completion rates, outcomes, measurement times, and information on the risk of bias. The data were arranged manually and tabulated using standardized forms including data from studies that fulfilled our requests for additional information.

### 2.4. Data Synthesis and Analysis

We stratified data into comparable subgroups for meta-analysis for each outcome: knowledge, attitude, and practice. Furthermore, we separated practice into two subgroups: self-reported practices and observed practices. As in similar cases [57,66], due to studies using different measurement instruments and scales, we calculated the Hedge’s *g* standardized mean differences (SMD) to measure the effect size, as proposed by Borenstein et al. [67]. Due to variation across studies, we conducted a random effect meta-analysis using Hedges’s *g* with a 95% confidence interval (CI) and the two-sided *p*-value for each outcome [67,68]. 

Heterogeneity among the studies in terms of effect measures was assessed using the I² statistic. This index can be interpreted as the percentage of total variability in a set of effect sizes due to true heterogeneity (between-studies variability) [69]. Higgins et al. 2003 suggested the use of I^2^ values of 25%, 50%, and 75% as low, moderate, and high, respectively [70]. Thus, an I^2^ value greater than 50% is indicative of substantial heterogeneity. We also assessed the evidence of risk of publication bias through a funnel plot and statistical tests, including Egger’s test [71] and the Begg’s test [72] (with a 95% confidence interval). We ran the meta-analysis in RStudio using the metafor package [73] and the meta package [74]. To reduce the risk of bias, two independent reviewers assessed each study. Randomized studies were assessed by using Cochrane’s tool RoB2 [75,76]. Here, the judgment criteria included 3 levels (low risk of bias, some concerns, or high risk of bias) for each of the 5 bias domains. Nonrandomized studies were assessed by using the ROBINS-I tool [77]; the judgment criteria included 5 levels (low, moderate, serious, critical, and no information) for each of the 7 bias domains [78]. The risk of bias visualization was done using robvis [79]. Finally, we summarized the findings reported in each study (Table 1).

## 3. Results

During the initial search, we found 1094 papers. Then, after removing duplicates, our database was reduced to 321 papers. Following data screening and the application of exclusion criteria, we removed 200 more studies. One hundred twenty-one studies underwent full-text review. However, after applying the inclusion criteria, only 31 papers were eligible for inclusion in the literature review (see Figure 1). We classified the 31 final papers into three categories based on their main outcomes: changes in knowledge, attitude, and practices toward food safety and hygiene following training interventions. Twenty-six of the 31 studies reported changes in knowledge, 12 discussed changes in attitude, and 16 reported changes in food safety practices. Regarding the publication rate, we found that food safety and hygiene training interventions seem to have increased since 2011. Regarding the country of origin, most of the studies were published in the United States (29%), followed by Malaysia (13%), and Canada, Brazil, and the United Kingdom, with equal proportions (6.5%), see Supplementary Tables S2 and S3. As for the research settings, the studies were conducted mainly in schools or universities (5/31), food process facilities (4/31), hospitals (4/31), restaurants (3/31), street food establishments or food trucks (3/31), farms/greenhouses (2/31), and multi-settings (2/31), among others.

Regarding the sample size, the studies varied from *n* = 10 to *n* = 194. There were 64 different interventions conducted among the 31 studies, with face-to-face/lectures (25/64) being the most frequent type of training intervention, followed by lectures combined with practice demonstrations (14/64), computer-based training (6/64), videos (4/64), videos combined with either a lecture (1/64) or a lecture and a demonstration (2/64), lectures combined with an incentive (1/64) or with demonstrations and incentives (2/64), and booklets (2/64), among others. We found that no studies used any kind of intervention involving social media. Regarding the type of study, eleven studies were pre-post studies, twelve relied on RCT, and eight performed a cross-sectional study with a trained group and a non-trained group. As for the measurement instruments, twelve studies administered surveys, one administered a test, two used checklists, and the rest did not report the used measurement instruments. Regarding gender, 13 papers reported that the majority of participants were female, while males represented the majority in nine studies, and one study had an equal proportion (50% of each). Eight studies did not report gender. The main outcomes, descriptions, statistics, and other relevant information of each study are summarized in Table 1.

**Figure 2:**
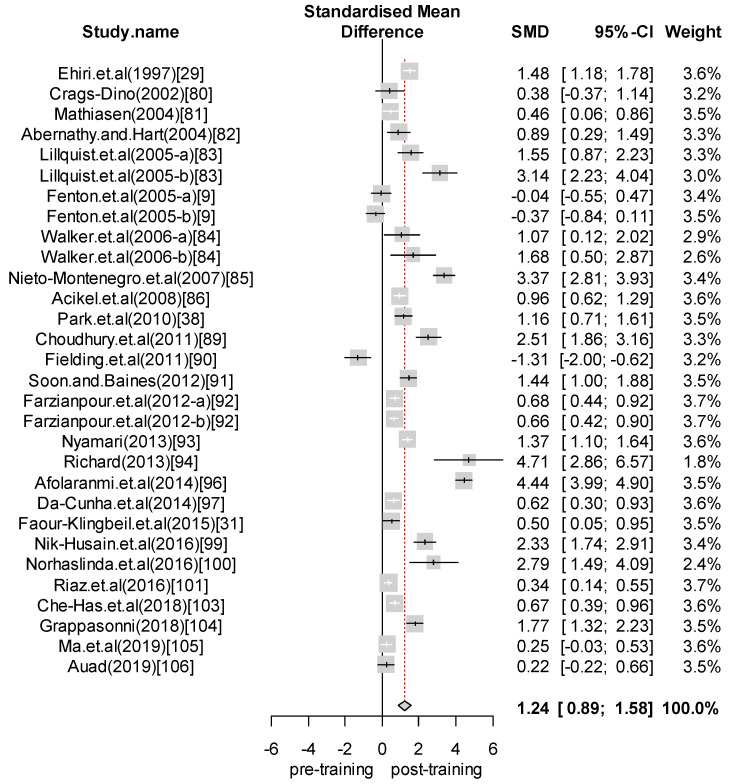
Forest plot—Knowledge.

**Figure 3:**
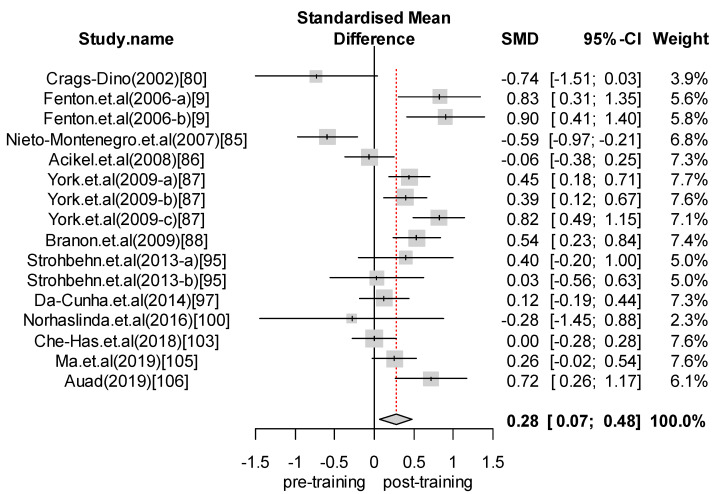
Forest plot—Attitude.

**Figure 4:**
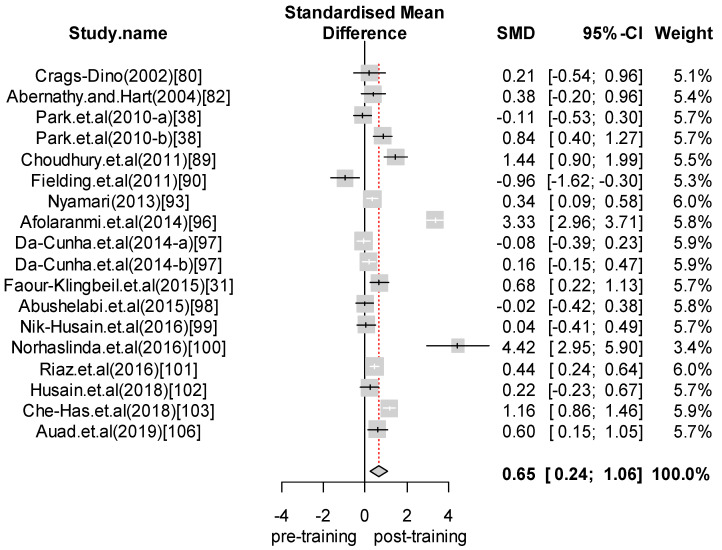
Forest plot—Overall practice.

**Figure 5:**
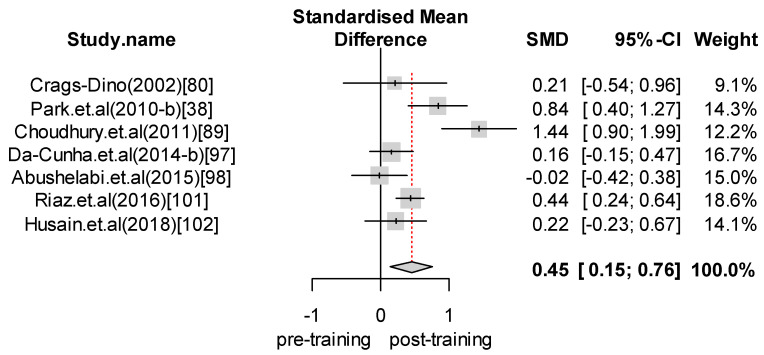
Forest plot—Observed practice.

**Figure 6:**
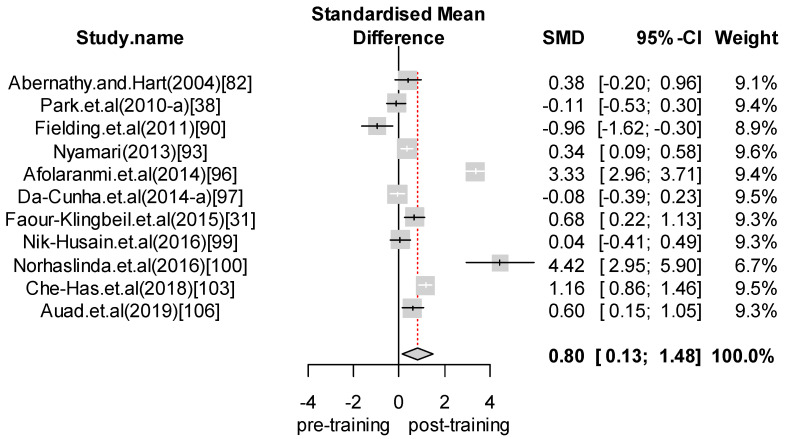
Forest plot—Self-reported practice.

We performed a meta-analysis of the effects of food safety training interventions on the KAP of food handlers. Overall, we found that food safety training interventions had a significant effect on knowledge changes, with an SMD of 1.24 (CI = 0.89 to 1.58; *p*-value = 0.0001). In relation to attitude, our analysis results indicate that food safety training has a positive effect, giving an SMD of 0.28 (CI = 0.07 to 0.48; *p*-value = 0.008) for the attitudes of food handlers toward food safety and hygiene. Finally, with respect to practice, the overall effect size was estimated to be SMD = 0.65 (CI = 0.24 to 1.06; *p*-value = 0.0018). For those interventions with self-reported practices, we found an effect size of SMD = 0.80 (CI = 0.13 to 1.48; *p*-value = 0.0201). In contrast, for studies reporting observed practices, the effect size was SMD = 0.45 (CI = 0.15 to 0.76; *p*-value = 0.0035). Figure 2, Figure 3, Figure 4, Figure 5 and Figure 6 show the forest plot for each outcome. Overall, food safety KAP was significantly higher as a result of training interventions. This phenomenon was particularly noticeable in the knowledge component. The forest plot in Figure 2 shows that most of the individual results lay close to 1. Such results strongly suggest that training increases knowledge of food safety and improves food safety attitudes and practices among food handlers.

We graphically assessed the risk of publication bias through funnel plots which, as the supplementary Figures S1–S5 depict, were symmetric. The null hypothesis for the Begg’s and Egger’s tests indicated an absence of bias in the selected studies. For knowledge, the Egger‘s test did not indicate any risk of publication bias, while the Begg’s test did indicate a moderate level of risk (i.e., Begg’s test: *p*-value = 0.044 and Egger’s test: *p*-value = 0.054); however, the data seem symmetric in the funnel plot (see Figure S1). As for the effects of food safety training on attitude changes, we also found no evidence of publication bias, both in tests and in the plot (Begg’s test: *p*-value = 0.653 and Egger’s test: *p*-value = 0.763). Finally, we found no statistical evidence of a risk of publication bias for the practice component (Begg’s test: *p*-value = 0.472 and Egger’s test: *p*-value = 0.608) and the graphic shows symmetry as well. In this review, the heterogeneity was considered high for knowledge (I^2^ = 95.3%), attitude (I^2^ = 77.7%), and for practice (I^2^ = 94.9%). Regarding the risk of bias for randomized studies, six studies were evaluated with some concerns of risk of bias, four studies with low risk, and two studies with high risk. For nonrandomized studies, ten were evaluated with moderate risk of bias, nine studies with serious risk, and none as low risk. The visualization data are shown in Supplementary Tables S4 and S5.

## 4. Discussion

This systematic review has summarized the effects of training interventions on the knowledge, attitudes, and practices of food handlers towards food safety and hygiene. Change in knowledge was assessed in 26 out of 31 studies; therefore, this was the most frequently reported outcome. This result is consistent with previous studies [58,107], and a significant amount of information is available, so it is probably easier to measure knowledge than attitude or practice. We found evidence that training interventions have a significant effect on increased knowledge toward food safety and hygiene across different type of settings such as fresh produce [91], food service operators [108], schools [80], restaurants [82], households [101], and multi-settings [97]. On the other hand, one study found no difference in knowledge between a control and an intervention group except for a positive attitude, so it can be considered to be optimistically biased [90]. This phenomenon has been demonstrated in previous research [90,109,110].

Attitude was assessed in 12 out of 31 studies, most of them assessing one intervention while some studies evaluated two [9,95] or three interventions [87]. Considering the summarized effect size, a SMD = 0.28 suggests a moderate effect for the positive attitude of food handlers; this is similar to previous studies [57,58,66]. Both studies [9,95] reported similar improvements in attitudes, either with face-to-face training or computer-based (CB) instruction. This is consistent with [84], who stated that participants learned equally well whether the instructional format was CB or instructor-led training. In addition, in studies where food handlers had attended food hygiene training previously [97,103], food safety attitude remained the same. According to our findings, most studies reporting an increase in knowledge also reported an increase in attitude [9,97,105,106]. However, an increase in knowledge might not necessarily bring about an improvement in attitude. This was the case for four studies [80,85,86,100]. The reason for this is unclear, yet some factors that could partially explain this could be length of the training [80], lack of repetition of the training [86], or previous hygiene enforcement program within the control group [85]. Attitude is a measure of the degree to which a person has a favorable or unfavorable evaluation of behavior [27]. In this regard, providing employees with training that does not promote a positive change with attitude [80], subjective norms, and perceptions of control may not contribute to improving intention (and ultimately behavior) to perform the behaviors [111].

Practice and behavior were measured in 16 studies, two of them assessing two outcomes (self-reported and observed practice) and the rest just one. The summarized effect of food safety training on practices showed that the interventions increased food safety practices, both for the 11 studies with self-reported practices and the seven studies with observed practices. Previous studies reported similar improvements, either self-reported or observed practices, but with a slightly smaller effect for the self-reported practices [38,97]; this consistent agreement between self-reported and observed behaviors was reported previously [23]. However, this is contrary to expected, since self-reported data are usually susceptible to social desirability bias [112], i.e., the tendency of respondents to give socially desirable responses in such a way as to be viewed favorably by others [113]. Thus, respondents tend to overestimate their food safety practices as being higher than their actual practices deserve [38,66,114,115]. On the other hand, observed practices could be affected by the “Hawthorne effect” where the changes in a person’s behavior may be due to the presence of an observer.

In this research, inconsistencies between self-reported and observed practices were detected by [106], with 95% being the self-reported rate of washing hands and 82.5% for keeping hair covered with a cap; however, the observations showed only 50% and 17.5% of compliance, respectively. For studies assessing practices thorough observations, evaluation was mainly done using a checklist [38,97,98,99,116].

The implementation of food safety and hygiene practices has the final objective of preventing foodborne illnesses. Food safety behaviors are often subdivided into specific behavioral constructs such as personal hygiene, adequate cooking of foods, avoiding cross-contamination, keeping foods at safe temperatures, and avoiding food from unsafe sources [117]. Behavior outcomes provide a more direct measure of intervention effectiveness compared to knowledge and attitudes [66]; however, food safety practices were measured in only 16 out of the 31 studies. This is consistent with the proportions reported by Viator et al. [107]. Moreover, an integrative review conducted by Zanin et al., [118] stated that 50% of the selected studies reported no translation of knowledge into attitudes/practices. In this review, we found evidence of close to 25% translation into both attitudes and practices. In addition, food safety practices of food handlers are associated with the type of management, i.e., tending to be higher in corporate-managed than owner-operated [31]. Incorporating practical assessment, such as observations, could help owner-operated organizations, since in some cases observation is more important than self-reported practices in order to represent actual behaviors [99,119].

### 4.1. Food Safety and Hygiene Training

Overall, all nine food safety training interventions that incorporated theory and practice (T&P) demonstrations were more effective in terms of knowledge gain than those that only incorporated theoretical training. This is consistent with [83], who found that training that incorporated active participation was more effective than traditional passive instruction. Nevertheless, those studies reporting T&P presented a poor improvement in attitude [85,86]. Finally, the seven and eleven interventions based on T&P and theory, respectively, showed similar practice improvement in 71% and 80% of the studies, respectively.

Although the ultimate goal is to prevent foodborne diseases, no study reported an impact on this goal. As expected, the results were based around the change in KAP as a mean to avoid food safety risk. Thus, theoretical training based on KAP is commonly used to improve handlers’ food safety performance [106]. However, some authors have reported flaws, mainly in the assumption that the received information is translated into practices and behaviors [100,103].

Food safety and hygiene are critical in all steps in the farm-to-fork chain. In an ideal scenario of the farm-to-fork continuum, a total absence of foodborne pathogens and opportunistic bacteria is obviously desired [120]. Nevertheless, despite good knowledge, attitude, and self-reported practices, there may be poor performance in hygiene [121] and food safety practices. Bacteria might exist in nature in a range of different metabolic stages, such as dormant, active, and growing; thus, it is important to detect bacteria and ascertain whether they are potentially active [120]. Despite the central role that food workers’ hands play in bacterial transfer among food and various surfaces [81], only one study assessed the number of bacteria growing on cultures obtained from the hands [86], while another demonstrated cross-contamination with hand hygiene sessions using GloGerm^®^ powder and UV light [91]. Both studies showed improved knowledge of food handlers. Similarly, it is well known that an effective way to control food poisoning is to maintain hygienic surroundings [103]. Thus, additional evaluations and inspections including surface cleanliness and hand cultures seem to be a suitable part of training [122]. Similarly, frequent practical and hands-on sessions will create a much more vivid experience for workers [83,89,91]. Active learning, e.g., a training session that raises awareness of the possibility that *E. coli* bacteria may accumulate under the fingernails should also demonstrate the correct handwashing procedure and require the learner to practice until he or she can successfully demonstrate effective performance of that procedure [85].

Also, risk perception acts as a guide for decisions about behavior and can be a barrier to following a particular activity or procedure or not [123]. In this regard, there are different approaches to food safety training. Some include cases of victims of food poisoning [91] during food safety training to connect with audiences’ lifestyles, incorporate fear, and enhance the perception of risk [58]. Moreover, to be effective, training programs should be based on appropriate adult education theory [124], the possibility of human error [125], and make sure that the reading comprehension level of the text is suitable for most food handlers [9]. Training programs that are more closely associated with a worksite are potentially more effective, especially if supported by practical reinforcement of the message [85,126].

The frequency [51] and length of exposure [127] for a training program are significative factors in the obtained outcome. For studies reporting the length of intervention, the majority were conducted in one day with a follow-up period between 2 and 8 weeks, with 1 year being the longest follow up period [82]. Moreover, because knowledge decreases over time [5], food safety and hygiene training should be provided frequently [51] to prevent the information from being forgotten and also to increase the level of knowledge [86]. Some studies suggest refresher retraining after 2 years [108] and before 5 years from initial certification [5]. For food establishments, we found that the educational level and professional training have significant effects on knowledge, practice [49,98], and food handlers’ positive attitudes [49,103]. However, the inclusion of adult education concepts, skill-based programs with interconnected sessions [85], and even the use of YouTube^®^ videos [91] can be effective for low literacy audiences. In this regard, farm employees with low educational attainment have also demonstrated significant knowledge gain [85,91].

Commitment and motivation from supervisors and management, as well as proper support and facilities given to staff are critical for the success of food safety and hygiene intervention. Training moves people in the right direction but not far enough [88]. In this regard, food handlers’ attitudes are significantly related to the management environment [31], thus supervisory support enforcement plays a significative role [85] in demonstrating and emphasizing the importance of following proper food safety practices [88], as well as being role models themselves [91]. Moreover, because transforming knowledge into behavior is complex, training from top management to all employees is crucial [128], inasmuch as successful food safety intervention must be based on firm theories [99]. Furthermore, additional key factors are the supervisors’ years of experience [5], clear responsibilities of food managers, and written agreement related to practicing sanitization procedures [99], as well as trained and certified managers helping to reduce critical food safety violations [129].

In terms of settings, most of the studies were carried out in restaurants and street food establishments, hospitals and schools, greenhouses and farms, and industrial food processing companies. This is in accordance with a previous study which found that the most frequently reported settings were restaurants and street food establishments [58]. In this context, the restaurant industry has been labeled as one of the most recurrent sources of foodborne illness outbreaks [130]. Therefore, food safety certification of kitchen managers appears to be a significant factor in outbreak prevention in restaurants [131]. A combination of inspection results with a mandatory training and certification program may mitigate food safety risks [132].

Many barriers and factors (environmental, social, cultural, belief systems, and so on) can affect whether food handlers effectively implement food safety practices in their workplaces [30,31,122,133], including a lack of adequate food safety training, time pressure, competing job tasks, lack of or inconvenient locations of equipment/resources, lack of managerial support, lack of motivation/incentive, lack of reminders, or lack of clarity in food safety messages [25,90,98,122,134,135,136]. As expected, studies from developing countries have experienced some fundamental barriers, including a lack of infrastructure, poor working conditions, ill-functioning equipment, a lack of water, and insufficient supervision [89,93]. Interestingly some studies from developed countries have experienced some limitations regarding literacy [94] and a potential language barrier [83], mainly because food handlers were not native speakers.

Regarding the training interventions among the selected studies, 27% were based on international guidelines (including WHO, HACCP, GMP, and ServSafe^®^), 18% on national guidelines, 18% on previous studies, and the remaining studies did not report this information. The guidelines vary by sector (restaurants, meat industry, dairy industry, etc.), legislation, or requirements of the country or region in which a company is located, market conditions, and certifications. Despite the frequent food-related incidents attaching great importance to the certification system [137], only 41% of the included studies awarded some national or international certification for food handlers. High costs could discourage companies from implementing certifications. In this sense, local governments should support organizations [137], mainly those that rarely invest in training or certification. A powerful way to win the interest of politicians and policy makers is to be able to attach a monetary value to food-related illness [138]. In this regard, the overall annual estimated cost of foodborne illness has remained relatively constant since 2005 at approximately GBP 1.5 billion in England and Wales and 152 billion USD in the USA [138]. Even though regulations and voluntary certifications are commonly thought of as driving forces to improve the safety and quality of food products [137], legislation might lead food handlers to undergo training only for certification without being motivated to acquire and use new knowledge [97]. A study found that the number of food safety violations did not differ as a function of certification [129]. Thus, certifications and legal requirements may not guarantee food safety [139].

### 4.2. Limitations

Our study has several major limitations. Firstly, differences in data (settings and data collection/processing approaches) and the multi-component nature of food safety and hygiene training makes it difficult to generalize the results. Second, most studies used observational pre–post designs. As a result, the absence of matched comparison groups, the potential presence of confounding variables, and the lack of randomization prevented the reported outcome improvements from being causally linked to the interventions. Third, the evaluation of KAP limited our ability to make conclusions about the behavior of the food handler. Fourth, knowledge, attitude, and practice are often subdivided into specific constructs; however, our ability to investigate these concepts in detail was limited by the availability and reporting of primary research, as many studies only reported overall scores or scales. Moreover, the determination of workers’ behavior using the self-reported technique before education was an important limitation in some included studies. Finally, there is a possibility that the “Hawthorne effect” led to the improvements reported in the studies.

## 5. Conclusions

Foodborne diseases continue to be a global problem, causing substantial morbidity and mortality and significant costs. According to our results, food safety and hygiene training have positive impacts on food handlers’ knowledge, attitude, and practice. Effective and frequent food safety training of food handlers continues to be an initial step in ensuring that food safety concepts are at least introduced. Despite knowledge being delivered by training, it cannot just be translated into desired changes in attitudes and practice. The inclusion of practical demonstration and continuous support might increase positive attitudes towards food safety and hygiene practices among food handlers with the ultimate goal of minimizing the incidence and prevalence of foodborne hazards. Moreover, effective food safety training should be relevant to the situation, promote active learning, increase risk perception, and consider the work environment. Because computer-based (CB) training was not found to differ from face-to-face training in terms of the outcome obtained, CB programs could be used more extensively, since they are an efficient and cost-effective way to educate staff.

In this regard, we identified several barriers to attaining proper food safety and hygiene practices, which should be considered by educators with appropriate adjustments according to the stage of the food supply chain, as well as the market, regional, and cultural characteristics. Similarly, training interventions should be based on international or national guidelines and adapted to different sectors, legislations, and certifications. Furthermore, local governments should support organizations, especially those that rarely invest in training and certification like SMEs, small farms, restaurants, or street food services. Finally, certifications and legal requirements may not guarantee food safety and hygiene, but when properly supported by resources, commitment, leadership, and a receptive management culture, food safety and hygiene practices may improve.

## Figures and Tables

**Table 1:** Summary of Findings.

Author, Year	Setting, Location	Main Intervention	Study Overview	Outcome	Summary of Findings Mean (SD)
Ehiri (1997) [29]	Centres and City Council, Scotland	Elementary food hygiene training	RCT; *nc*1 = 75, *nc*2 = 204; *ni*1 = 94, *ni*2 = 94; participants = 63% female	Knowledge score	Control = 61.66; Intervention = 66; (*p* < 0.05)
Craggs-Dino (2002) [80]	Foodservice at schools, USA	Food safety videotapes	RCT; *nc* = 10, *ni* = 22; TL = 3 h; FU = 2 weeks; participants = 94% female	Knowledge score	Control Pre 57.7 (14.7), Post 60.0 (12.3); Intervention Pre 53 (14.8), Post 65.3 (14.0)
				Attitude score	Control Pre 4.0 (0.5), Post 4.38 (0.3); Intervention Pre 4.12 (0.5), Post 4.04 (0.5)
				Observed behavior (practice)	Control Pre 4.39 (0.4), Post 4.30 (0.3); Intervention Pre 4.44 (0.4), Post 4.38 (0.4)
Mathiasen (2004) [81]	Greenhouses, Canada	Agricultural training video	Pre-post study; *n* = 50; TL = 1 day; participants = 78.3% female	Knowledge score	Pre 4.56 (0.312), Post 4.68 (0.165); (*p* = 0.07)
Abernathy and Hart (2004) [82]	Restaurants, Canada	Critical Approach© program.	RCT; *nc* = 42, *ni* = 16; FU = 1 year	Knowledge quantity	Control Pre 36.5 (28.6), Post 49.2 (36.4); Intervention Pre 50 (41), Post 81.3 (32.5)
				Self-reported practice score	Control Pre 46.8 (38.8), Post 57.1 (47.8); Intervention Pre 56.3 (48.8), Post 75(43.3)
Lillquist et al. (2005) [83]	Food handlers, USA	Lecture and video	RCT; *n* = 22; TL = 1.5 h; FU= 2 weeks; participants = 82% female	Knowledge score	Control 8.00 (3.2); Intervention 12.4 (2.3); (*p* <0.050)
		Lecture, video, and hand washing training	RCT; *n* = 22; TL = 1.5 h; FU= 2 weeks	Knowledge score	Control 8.00 (3.2); Intervention 15.80 (1.3); (*p* < 0.05)
Fenton et al. (2006) [9]	Food processing facilities, USA	Face to face training	RCT; *nc* = 28, *ni* = 31; TL= 1 day	Knowledge score	Control Pre 15.1 (1.9), Post 15.8 (2.1); Intervention Pre 12.60 (3.8), Post 15.70 (3.0); (*p* < 0.05)
				Attitude score	Control 40.3 (2.5); Intervention Pre 36.40 (5.6), Post 40.30 (3.4); (*p* < 0.05)
		Computer based training	RCT; *nc* = 28, *ni* = 35	Knowledge score	Control Pre 15.1 (1.9), Post 15.8 (2.1); Intervention Pre 12.80 (4.4), Post 14.5 (4.5); (*p* < 0.05)
				Attitude score	Control 40.3 (2.5) Intervention Pre 36.50 (5.3), Post 40.60 (3.5); (*p* < 0.05)
Walker et al. (2006) [84]	Care settings for elderly residents, USA	Computer based training	Pre-post study, *n* = 10; TL = 40 min	Knowledge score	Pre 25.9 (2.85), Post 28.6 (1.89)
		Instructor-led workshops	Pre-post study, *n* = 8 TL = 62 min	Knowledge score	Pre 23.88 (3.18), Post 28.25 (1.39)
Nieto-Montenegro et Al. (2007) [85]	Mushrooms companies, USA	Visual aids, discussion topics, demonstrations, and hands-on activities	RCT; (Track C packing house); *nc* = 52, *ni* = 61; TL = 225 min; participants = 49.1% female	Knowledge score	Control Pre 6.79 (1.55), Post 6.16 (0.166); Intervention Pre 5.84 (1.43), Post 9.29 (0.163)
				Attitude score	Control Pre 87.1 (6.3), Post 97.4 (2.2); Intervention Pre 41.30 (19.7), Post 94.5 (6.3)
Acikel et Al. (2008) [86]	Kitchen of a military medical academic, Turkey	Lecture and practice	Pre-post study; *n* = 78; TL = 1 day; FU = 1 mo.; participants = 14% female	Knowledge score	Pre 45.60 (11.2), Post 56.50 (11.5); (*p* = 0.001)
				Attitude score	Pre 2.70 (1.73), Post 2.59 (1.8); (*p* > 0.05)
York et al. (2009) [87]	Restaurants, USA	Servsafe^®^ Training	RCT; *nc* = 140, *ni* = 94; TL = 4 h	Attitude score	Control 37.05 (23.86); Training group 47.77 (24.13)
		A theory-based intervention	RCT; *nc* = 140, *ni* = 83; TL = 4 h	Attitude score	Control 37.05 (23.86); Training group 46.38(23.04)
		Servsafe^®^ and a theory-based intervention	RCT; *nc* = 140, *ni*= 51; TL = 4 h	Attitude score	Control 37.05 (23.86); Training group 56.19 (21.45)
Brannon et al. (2009) [88]	University, USA	Formal food certification class	CS; *nc*= 68; *ni* = 120; participants = 68.5% female	Attitude score	No experience 8.78 (3.21); Well-informed 10.54 (3.3)
Park et Al. (2010) [38]	Restaurants, Korea	Lecture and demonstration techniques	Control-intervention (non randomized) and pre-post study; *ni* = 41, *nc* = 49; TL = 1 h; participants = 65.5% female	Knowledge score	Intervention Pre 49.30 (19.5), Post 66.60 (16.5); Control Pre 51.7 (17.4), Post 45.8 (18.8)
				Self-reported practice score	Intervention Pre 103.2 (14.7), Post 102.40 (16.4); Control Pre 107.5 (20.4), Post 105.4 (32.2)
				Observed practice score	Pre 57.2 (7.8), Post 63.7 (7.6)
Choudhury (2011) [89]	Street food vendors, India	Charts, posters, videos, role plays, demonstration, puppet shows, and handouts	Pre-post study; *n*1 = 43, *n*2 = 26; TL = 60 h	Knowledge score	Pre 22.30 (16.46), Post 63.50 (15.83); (*p* = 0.00)
				Observed practice score	Pre 4.57 (12.18), Post 44.67 (42.14); (*p* = 0.00)
Fielding (2011) [90]	Micro and SME Manufacturers of soups and sauces, UK	Landscape booklet	Control-intervention study (non randomized); *ni* = 26, *nc* = 16; TL = 6 weeks	Knowledge score	Control 91.23 (4.89); Intervention 85.22 (4.27)
				Self-reported practice score	Control 71.98 (9.62); Intervention 63.99 (7.12)
Soon and Baines (2012) [91]	Fresh produce farms, UK	Booklet, slides, youtube^®^, and demonstrations	Pre-post study; *n*1 = 62, *n*2 = 42; FU = 1 day; participants = 45% female	Knowledge score	Pre 5.74 (1.77), Post 7.76 (0.43); (*p* < 0.001)
Farzianpour (2012) [92]	Food preparation and supply centers, Iran	Face-to-face training	RCT; *nc* = 140, *ni* = 135	Knowledge score	Control Pre 39.81 (13.06), Post 42.62 (13.16); Intervention Pre 41.98 (15.51), Post 51.04 (11.51)
		Distant learning using educational booklet	RCT; *nc* = 140, *ni* = 145	Knowledge score	Control Pre 39.81 (13.06), Post 42.62 (13.16); Intervention Pre 40.89 (12.54), Post 51.88 (14.67)
Nyamari (2013) [93]	Hospitals, Kenya	Lectures, demonstration, group discussions, and practical experiences	RCT; *nc* = 140, *ni* = 129; TL = 1 week; FU= 3 mo.; participants = 54.8% female	Knowledge score	Control Pre 51.8 (15.1), Post 53.4 (17.8); Intervention 50.6 (16.5), 76.4 (15.5)
				Self-reported practice score	Control Pre 110.7, Post 102.7; Intervention Pre 101.3 (11.6), Post 105.3 (12.2)
Richard (2013) [94]	Delicatessens meat shops, USA	Face to face training	RCT; *nc* = 10, *ni*=10; FU= 6 months; participants = 20.7% female	Knowledge score	Control 0.6(0.7) Intervention 4.3(0.8)
Strohbehn (2013) [95]	Retail foodservices (restaurants, hospitals, schools, others), USA	Face to face Training	Pre-post study; n1= 39, *n*2 = 21; participants = 80.9% female	Score in communicating safety culture attitude	Pre 3.99 (0.32), Post 4.13 (0.41)
		Computer based instruction	Pre-post study; *n*1 = 39, *n*2 = 15; participants = 50% female	Score in communicating safety culture attitude	Pre 3.99 (0.32), Post 4.0 (0.29)
Afolaranmi (2014) [96]	Kitchens of Secondary schools, Nigeria	Lectures, practical demonstrations, charts, manuals, and posters	Pre-post study; *n*1 = 132, *n*2 = 130; TL = 1 day; FU = 3 mo.; participants = 93.2% female	Knowledge score	Pre 8.91 (2.18), Post 22.20 (3.62); (*p* <0.001)
				Self-reported practice score	Pre 32.66 (3.24), Post 44.46 (3.80); (*p* < 0.001)
da Cunha (2014) [97]	Street food kiosks, beach kiosks, restaurants, hospitals and school, Brazil	Lecture based training	CS; *nc* = 58, *ni* = 125; FU = 10 h; participants = 65.5% female	Knowledge score	Untrained 5.40 (1.8); Trained 6.60(2.0); (*p* <0.001)
				Attitude score	Untrained 9.00(1.1); Trained 9.20 (1.8); (*p* = 0.40)
				Self-reported practice score	Untrained 36.00 (3.6); Trained 35.70 (4.0); (*p* = 0.59)
				Observed practice score	Untrained 23.00 (6.25) Trained 24.20 (7.8); (*p* = 0.67)
Faour-Klingbeil (2015) [31]	Food businesses, Lebanon	Face to face/lectures	CS; *nc* = 46, *ni* = 34; participants = 93% female	Knowledge score	Control 52.20 (19.6); Intervention 62.50 (21.7)
				Self-reported practice score	Control 57.60 (14.3); Intervention 66.40 (10.7); (*p* < 0.01)
Abushelaibi (2015) [98]	Food stablishments, UAE	Face to face training	Pre-post study; *n* = 48; FU = 3 months	Observed practice score	Pre 2.80 (0.27), Post 2.79 (0.23); (*p* < 0.05)
Nik Husain (2016) [99]	School canteens, Malaysia	Health talks, demonstrations, self-practice, posters	RCT; *nc* = 46, *ni* = 33; TL = 235 min; FU = 3 weeks; participants = majority female	Knowledge score	Control 19.49 (0.57) Intervention 21.03 (0.76); (*p* = 0.59)
				Self-reported practice score	Control 28.59 (5.45); Intervention 28.79 (4.51)
Norhaslinda (2016) [100]	Hospitals, Malaysia	Training course attendance	CS; *ni* = 47, *nc* = 3; participants = 72% female	GMP knowledge score	Untrained 79.00 (2.51); Trained 83.30 (1.46)
				GMP attitude score	Untrained 88.00 (3.0); Trained 87.00 (3.49); (*p* = 0.65)
				Self-reported practice score	Untrained 83.40 (2.08); Trained 91.20 (1.72); (*p* = 0.74)
Riaz (2016) [101]	Household, Bangladesh	Face to face workshops (courtyard counselling meetings)	Pre-post study; *n* = 194; TL = 4 days; participants = 100% female	Knowledge score	Pre 23.70 (5.7), Post 25.60 (5.3); (*p* < 0.001)
				Observed practice score	Pre 20.50 (5.34), Post 22.10 (3.9); (*p* < 0.001)
Nik Husain (2018) [102]	School canteens, Malaysia	Demonstrations, self-practice, and posters	RCT; *ni* = 33, *nc* = 46; TL = 195 min; participants = 89.9% female	Observed handwashing practice score	Control Pre 35.06 (29.23), Post 7.59 (29.84); Intervention Pre 29.0 (24.17), Post 44.52 (31.97)
Che-Has (2018) [103]	Food stablishment, Malaysia	Face to face training	Pre-post study; *n* = 100; Participants = 48% female	Knowledge score	Pre 11.12 (2.69), Post 12.83 (2.36); (*p* = 0.00)
				Attitude score	Pre 4.21 (0.54), Post 4.21 (0.54); (*p* = 1.00)
				Self-reported practice score	Pre 9.01 (1.09), Post 10.22 (0.99); (*p* = 0.00)
Grappasonni (2018) [104]	board merchant ships, Italy	Food safety training	CS; *nc* = 28, *ni* = 130; participants = male 100%	Knowledge score	Untrained 46.1 (3.4); Trained 52.9 (3.9)
MA (2019) [105]	Street vendors, China	Food safety training	CS; *nc* = 67, *ni* = 30; participants = 47.1% female	Knowledge score	Untrained 56 (16); Trained 60 (16); (*p* = 0.287)
				Attitude score	Untrained 59 (16); Trained 63 (15)
Auad (2019) [106]	Food trucks, Brazil	Food safety training	CS; *nc* = 18, *ni* = 22; participants = 20% female	Knowledge score	Untrained 7.00 (2.14); Trained 7.41 (1.44); (*p* = 0.638)
				Attitude score	Untrained 6.39 (1.29); Trained 7.23 (1.02); (*p* = 0.033)
				Mean score in self-reported practice	Untrained 7.28 (1.41); Trained 8.09 (1.27); (*p* = 0.085)

**Note.** SD indicates standard deviation; RCT, Randomized control trials; CS, Cross-sectional studies; TL, training length; FU, follow up; GMP, good manufacturing practices; *n*, sample; *nc*, control group sample; *ni*, intervention group sample; mo., Months; h, Hours; min, Minutes. The last name of the main author and the publication year are shown.

## Supplementary Materials

The following are available online at https://www.mdpi.com/2304-8158/9/9/1169/s1, Table S1: PRISMA checklist, Data S1: Search strategy, Table S2: Geographical distribution of studies selected, Table S3: Distribution per year of studies selected, Figure S1: Funnel plot for knowledge, Figure S2: Funnel plot for attitude, Figure S3: Funnel plot for overall practice, Figure S4: Funnel plot for self-reported practice, Figure S5: Funnel plot for observed practice, Table S4: Risk of bias for randomized studies, and Table S5: Risk of bias for nonrandomized studies.
