# Supplementary material for: A Systematic Review and Meta-Analysis of the Effects of Food Safety and Hygiene Training on Food Handlers
Source: Foods. 2020 Aug 25;9(9):1169. doi: 10.3390/foods9091169 (PMC7555000; doi:10.3390/foods9091169)
Supplement: Supplementary file 1 [file foods-09-01169-s001.pdf]

Table S1. PRISMA Checklist

Section/topic	#	Checklist item	Reported on section
**TITLE**			
Title	1	Identify the report as a systematic review, meta-analysis, or both.	Title
**ABSTRACT**			
Structured summary	2	Provide a structured summary including, as applicable: background; objectives; data sources; study eligibility criteria, participants, and interventions; study appraisal and synthesis methods; results; limitations; conclusions and implications of key findings; systematic review registration number.	Abstract
**INTRODUCTION**			
Rationale	3	Describe the rationale for the review in the context of what is already known.	Introduction
Objectives	4	Provide an explicit statement of questions being addressed with reference to participants, interventions, comparisons, outcomes, and study design (PICOS).	Introduction
**METHODS**			
Protocol and registration	5	Indicate if a review protocol exists, if and where it can be accessed (e.g., Web address), and, if available, provide registration information including registration number.	Materials and Methods
Eligibility criteria	6	Specify study characteristics (e.g., PICOS, length of follow-up) and report characteristics (e.g., years considered, language, publication status) used as criteria for eligibility, giving rationale.	Materials and Methods
Information sources	7	Describe all information sources (e.g., databases with dates of coverage, contact with study authors to identify additional studies) in the search and date last searched.	Materials and Methods
Search	8	Present full electronic search strategy for at least one database, including any limits used, such that it could be repeated.	Data S1
Study selection	9	State the process for selecting studies (i.e., screening, eligibility, included in systematic review, and, if applicable, included in the meta-analysis).	Materials and Methods
Data collection process	10	Describe method of data extraction from reports (e.g., piloted forms, independently, in duplicate) and any processes for obtaining and confirming data from investigators.	Materials and Methods
Data items	11	List and define all variables for which data were sought (e.g., PICOS, funding sources) and any assumptions and simplifications made.	Materials and Methods
Risk of bias in individual studies	12	Describe methods used for assessing risk of bias of individual studies (including specification of whether this was done at the study or outcome level), and how this information is to be used in any data synthesis.	Materials and Methods
Summary measures	13	State the principal summary measures (e.g., risk ratio, difference in means).	Materials and Methods
Synthesis of results	14	Describe the methods of handling data and combining results of studies, if done, including measures of consistency (e.g., I^2) for each meta-analysis.	Materials and Methods

Section/topic	#	Checklist item	Reported on page #
Risk of bias across studies	15	Specify any assessment of risk of bias that may affect the cumulative evidence (e.g., publication bias, selective reporting within studies).	Methods
Additional analyses	16	Describe methods of additional analyses (e.g., sensitivity or subgroup analyses, meta-regression), if done, indicating which were pre-specified.	N/A
**RESULTS**			
Study selection	17	Give numbers of studies screened, assessed for eligibility, and included in the review, with reasons for exclusions at each stage, ideally with a flow diagram.	Results and Figure 1
Study characteristics	18	For each study, present characteristics for which data were extracted (e.g., study size, PICOS, follow-up period) and provide the citations.	Table 1
Risk of bias within studies	19	Present data on risk of bias of each study and, if available, any outcome level assessment (see item 12).	Table S4
Results of individual studies	20	For all outcomes considered (benefits or harms), present, for each study: (a) simple summary data for each intervention group (b) effect estimates and confidence intervals, ideally with a forest plot.	Table 1 and Figures 2-6
Synthesis of results	21	Present results of each meta-analysis done, including confidence intervals and measures of consistency.	Results and Figures 2-6
Risk of bias across studies	22	Present results of any assessment of risk of bias across studies (see Item 15).	Results and Figures 7-9
Additional analysis	23	Give results of additional analyses, if done (e.g., sensitivity or subgroup analyses, meta-regression [see Item 16]).	N/A
**DISCUSSION**			
Summary of evidence	24	Summarize the main findings including the strength of evidence for each main outcome; consider their relevance to key groups (e.g., healthcare providers, users, and policy makers).	Results
Limitations	25	Discuss limitations at study and outcome level (e.g., risk of bias), and at review-level (e.g., incomplete retrieval of identified research, reporting bias).	Discussion
Conclusions	26	Provide a general interpretation of the results in the context of other evidence, and implications for future research.	Conclusions
**FUNDING**			
Funding	27	Describe sources of funding for the systematic review and other support (e.g., supply of data); role of funders for the systematic review.	N/A

Data S1. Search Strategy

1. Pubmed

Population

(Food?handl*[tiab] OR “food workers”[tiab] OR “food employees”[tiab]) AND (“food industry/process industry”[Mesh] OR food?manufactur* OR agri?food OR “farm” OR “restaurant” OR “retail” OR “food service” OR “food street” OR “food business” OR “hospital” OR “school” OR universit*)

Intervention

(“Intervention” OR “training”[tiab] OR “evaluation”[tiab]) AND ((“food safety”[tiab]) OR (“good manufacturing practices”[tiab]) OR (“hazard analysis”[tiab]) OR (hand?washing[tiab]) OR (person?l?hygiene[tiab]) OR (clean*) OR (sanitization) OR (cross?contamination) OR (temperature?control) OR (food?handl*))

Outcome

(“Knowledge”[tiab] OR “attitude”[tiab] OR “practice”[tiab] OR “behavior”[tiab]) AND (“food safety” OR “good manufacturing practices” OR “good hygiene practices” OR “hazard analysis” OR “foodborne diseases” OR hand?washing OR “personal hygiene” OR clean* OR “sanitization” OR cross?contamination OR “temperature control”)

2. The Cochrane Controlled Register of Trials (CENTRAL

Population

((Food handl*). ti,ab. OR (food workers). ti,ab. OR (food employees). ti,ab.) AND ((food industry OR food process industry)OR food?manufactur* OR agri?food OR farm OR restaurant OR retail OR food service OR food street OR food business OR hospital OR school OR universit*)

Intervention

(((Intervention). ti,ab. OR (training). ti,ab. OR (evaluation). ti,ab.) AND ((food safety). ti,ab. OR (good manufacturing practices). ti,ab. OR (hazard analysis). ti,ab. OR (hand?wash*). ti,ab. OR (person?l hygiene). ti,ab. OR (clean*) OR (sanitization) OR (cross?contamination) OR (temperature?control) OR (food handl*)))

Outcome

(((Knowledge). ti,ab. OR (attitude). ti,ab. OR (practice). ti,ab. OR (behavior). ti,ab) AND (“food safety” OR “good manufacturing practices” OR “good hygiene practices” OR “hazard analysis” OR “foodborne diseases” OR hand?washing OR “personal hygiene” OR clean* OR “sanitization” OR cross?contamination OR “temperature control”))

3. Ebsco

Population

(Food?handl*[tiab] OR “food workers”[tiab] OR “food employees”[tiab]) AND (“food industry/process industry”[Mesh] OR food?manufactur* OR agri?food OR “farm” OR “restaurant” OR “retail” OR “food service” OR “food street” OR “food business” OR “hospital” OR “school” OR universit*)

Intervention

(“Intervention” OR “training”[tiab] OR “evaluation”[tiab]) AND ((“food safety”[tiab]) OR (“good manufacturing practices”[tiab]) OR (“hazard analysis”[tiab]) OR (hand?washing[tiab]) OR (person?l?hygiene[tiab]) OR (clean*) OR (sanitization) OR (cross?contamination) OR (temperature?control) OR (food?handl*))
Outcome
(“Knowledge”[tiab] OR “attitude”[tiab] OR “practice”[tiab] OR “behavior”[tiab]) AND (“food safety” OR “good manufacturing practices” OR “good hygiene practices” OR “hazard analysis” OR “foodborne diseases” OR hand?washing OR “personal hygiene” OR clean* OR “sanitization” OR cross?contamination OR “temperature control”)

4. **Scopus**

Population
((“Food handlers”) AND (“food industry” OR “food process industry” OR “agri-food” OR “farm” OR “restaurant” OR “food street” OR “hospital” OR “school” OR “university”))
Intervention
((“Intervention” OR “training” OR “evaluation”) AND (“food safety” OR “hand washing” OR “personal hygiene” OR “clean and sanitization” OR “cross-contamination” OR “temperature control” OR “food handling”))
Outcome
((“Knowledge” OR “attitude” OR “practice” OR “behavior”) AND (“food safety” OR “foodborne diseases” OR hand washing OR “personal hygiene” OR “clean and sanitization” OR “cross-contamination” OR “temperature control”))

5. **Web of Science**

Population
((“Food handlers”) AND (“food industry” OR “food process industry” OR “agri-food” OR “farm” OR “restaurant” OR “food street” OR “hospital” OR “school” OR “university”))
Intervention
((“Intervention” OR “training” OR “evaluation”) AND (“food safety” OR “hand washing” OR “personal hygiene” OR “clean and sanitization” OR “cross-contamination” OR “temperature control” OR “food handling”))
Outcome
((“Knowledge” OR “attitude” OR “practice” OR “behavior”) AND (“food safety” OR “foodborne diseases” OR hand washing OR “personal hygiene” OR “clean and sanitization” OR “cross-contamination” OR “temperature control”))

Table S2. Geographical distribution of studies selected

Country	Total
**USA**	9
**Malaysia**	4
**Canada**	2
**Brazil**	2
**UK**	2
**Turkey**	1
**Korea**	1
**India**	**1**
**Nigeria**	1
**Lebanon**	1
**Bangladesh**	1
**UA Emirates**	1
**China**	1
**Scotland**	1
**Iran**	1
**Kenya**	1
**Italy**	1

Table S3. Distribution per year of studies selected

Year	Total
**1997**	1
**2002**	1
**2004**	2
**2005**	1
**2006**	2
**2007**	1
**2008**	1
**2009**	2
**2010**	1
**2011**	2
**2012**	2
**2013**	3
**2014**	2
**2015**	2
**2016**	3
**2018**	3
**2019**	2

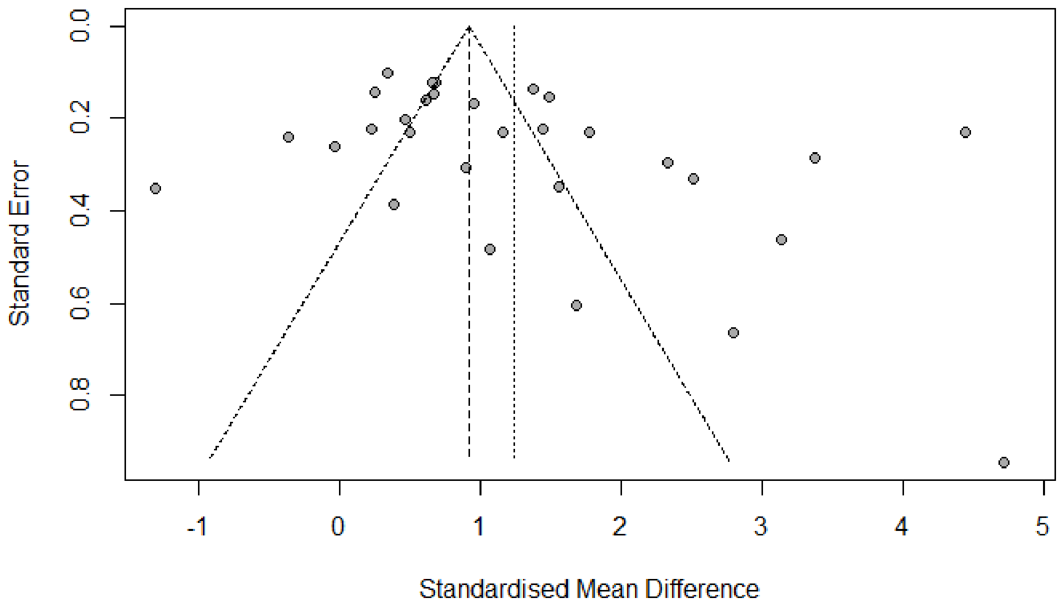

Figure S1. Funnel plot – Risk of publication bias of knowledge

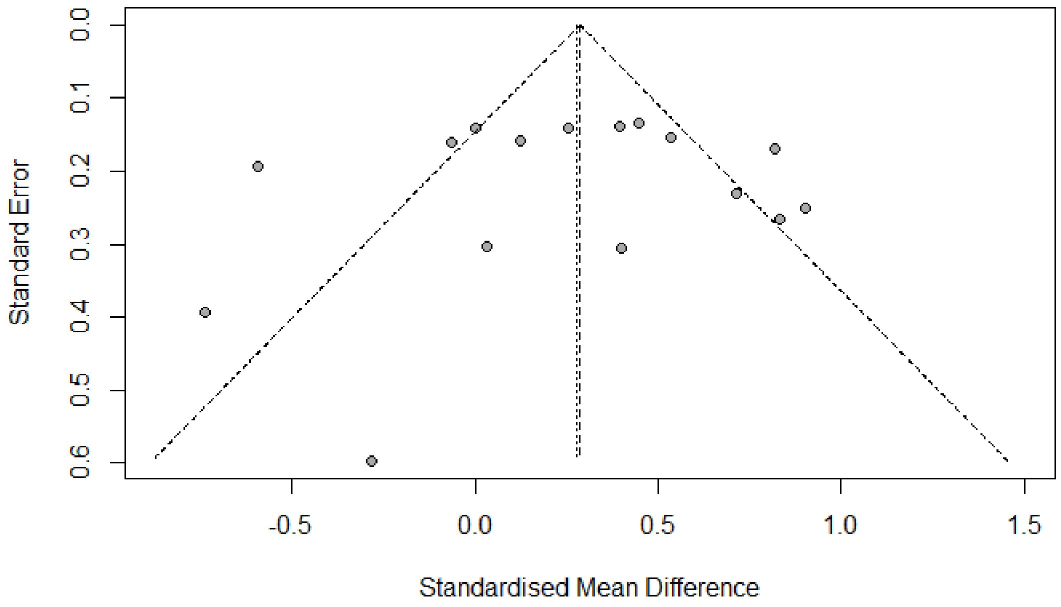

Figure S2. Funnel plot – Risk of publication bias of attitude

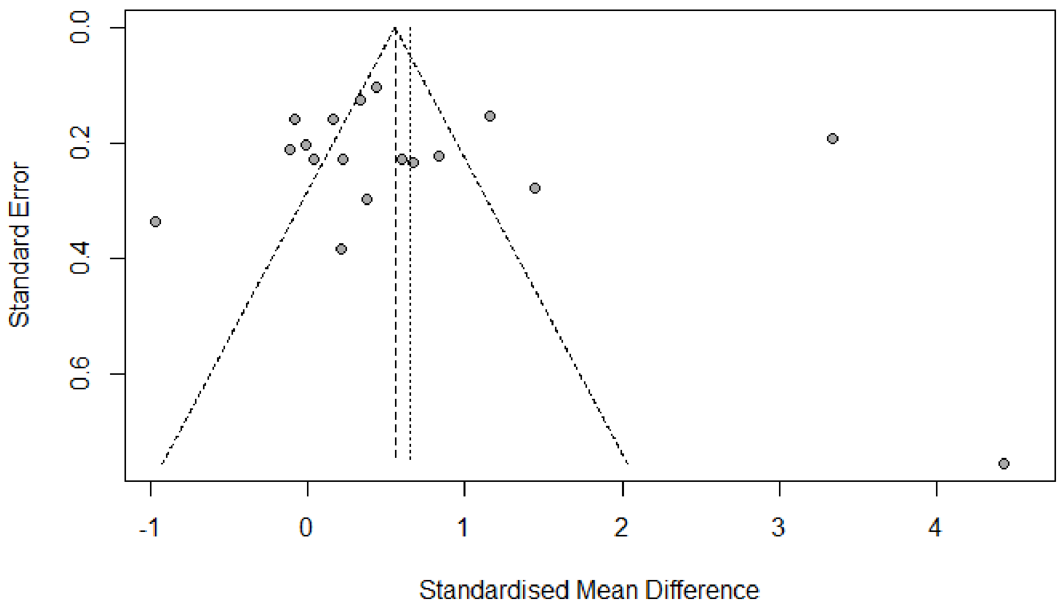

Figure S3. Funnel plot – Risk of publication bias of overall practices

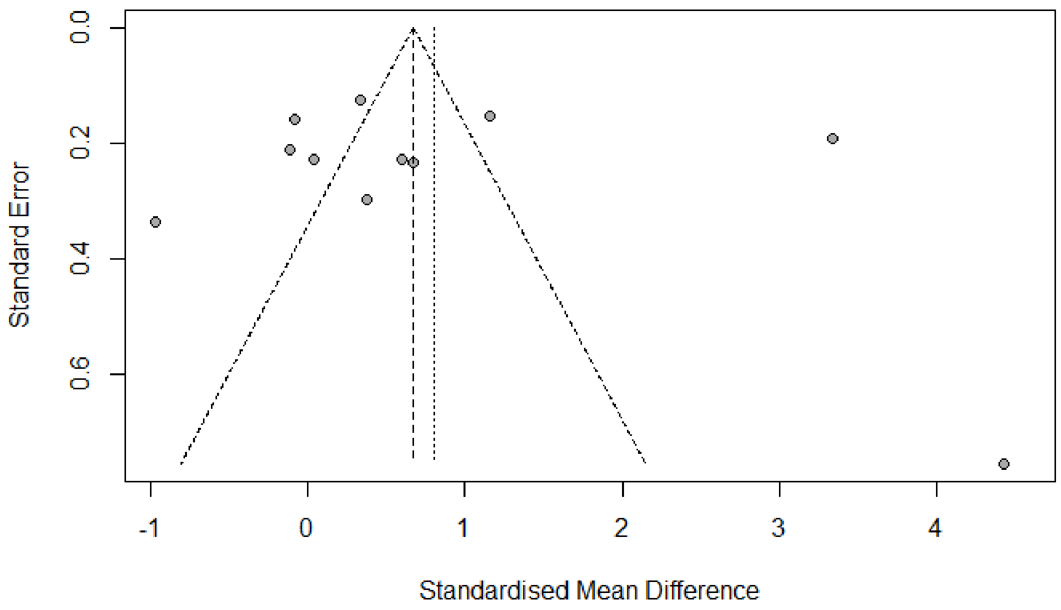

Figure S4. Funnel plot –Risk of publication bias of self-reported practice

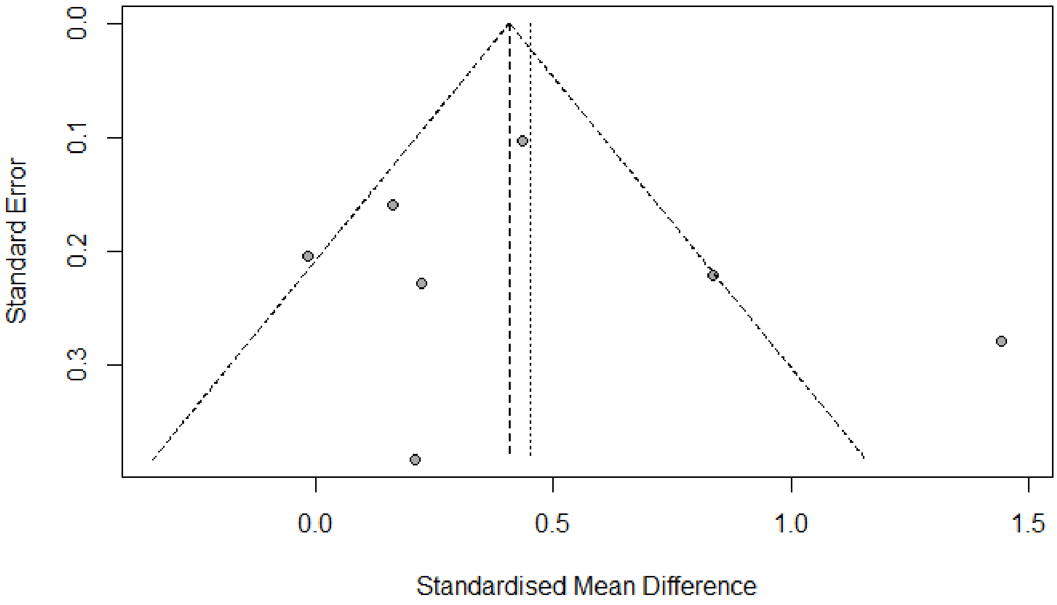

Figure S5. Funnel plot –Risk of publication bias of observed practice

Table S4. Risk of bias for randomized studies

Study	D1	D2	D3	D4	D5	Overall
Ehiri et al., 1997	+	+	+	+	+	+
Crags-Dino, 2002	+	-	+	+	+	-
Abernathy & Hart, 2004	+	+	+	+	+	+
Lillquist et al, 2005	+	-	+	+	+	-
Fenton et al, 2006	+	-	+	+	+	-
Nieto-Montenegro et al, 2007	+	+	+	+	+	+
York et al, 2009	+	+	+	+	+	+
Farzianpour et al., 2012	+	-	+	+	+	-
Richard, 2013	+	-	+	+	+	-
Nyamari, 2013	+	-	+	+	+	-
Nik Husain et al, 2016	+	X	+	+	+	X
Nik Husain et al, 2018	+	X	+	+	+	X

Risk of bias domains

Domains:
D1: Bias arising from the randomization process
D2: Bias due to deviations from intended intervention.
D3: Bias due to missing outcome data.
D4: Bias in measurement of the outcome.
D5: Bias in selection of the reported result.

Judgement
X High
- Some concerns
+ Low

Table S5. Risk of bias for nonrandomized studies

Study	D1	D2	D3	D4	D5	D6	D7	Overall
	Risk of bias domains							
Mathiasen, 2004	-	X	-	X	+	-	-	X
Walker et al., 2006	-	X	-	-	+	+	-	-
Acikel et al, 2008	-	X	-	-	+	-	-	-
Park et al, 2009	-	+	+	+	+	-	-	-
Brannon et al., 2009	-	X	-	X	+	+	-	X
Choudhury et al, 2011	-	X	-	+	+	-	-	-
Fielding et al, 2011	-	+	+	+	+	-	-	-
Soon and Blaines, 2012	-	X	-	+	+	+	-	-
Strohbehn et al., 2013	-	X	-	+	+	+	-	-
Afolaranmi et al, 2014	-	X	-	+	+	-	-	-
Da-Cunha et al, 2014	-	X	-	X	+	X	-	X
Faour-Klingbeil et al, 2015	-	X	-	X	+	X	-	X
Abushelaibi et al, 2015	-	X	-	+	+	-	-	-
Riaz et al, 2016	-	X	-	+	+	-	-	-
Norhaslinda et al, 2016	-	X	-	X	+	-	-	X
Has et al, 2018	-	X	-	+	+	-	-	X
Grasppasonniet al, 2018	-	X	-	X	+	-	-	X
Auad et al., 2019	-	X	-	X	+	-	-	X
Ma et al, 2019	-	X	-	X	+	-	-	X

Domains:
D1: Bias due to confounding.
D2: Bias due to selection of participants.
D3: Bias in classification of interventions.
D4: Bias due to deviations from intended interventions.
D5: Bias due to missing data.
D6: Bias in measurement of outcomes.
D7: Bias in selection of the reported result.

Judgement
X Serious
- Moderate
+ Low
